# Influence of phenolic compounds on color formation at different stages of the VHP sugar manufacturing process

**DOI:** 10.1038/s41598-022-24455-4

**Published:** 2022-11-19

**Authors:** Mirelle Márcio Santos Cabral, Yeda Medeiros Bastos de Almeida, Samara Alvachian Cardoso Andrade, Celso Silva Caldas, Jonnathan Duarte de Freitas, Clara Andrezza Crisóstomo Bezerra Costa, João Inácio Soletti

**Affiliations:** 1 Laboratório de Processos Industriais, Instituto Federal de Alagoas, Rodovia Joaquim Gonçalves s/n, Penedo, AL CEP 57200-000 Brazil; 2 Departamento de Engenharia Química, Universidade Federal de Pernambuco, Av. dos Economistas s/n, Recife, PE CEP 50740-590 Brazil; 3 Departamento de Química, Instituto Federal de Alagoas, Rua Barão de Atalaia s/n, Maceió, AL 57020-510 Brazil; 4 Departamento de Engenharia Química, Universidade Federal de Alagoas, Av. Lorival Melo Mota s/n, Maceió, AL CEP 57072-900 Brazil

**Keywords:** Chemistry, Engineering

## Abstract

Phenolic compounds are natural dyes contained in sugarcane juice and represent an important parameter in industrial processing, as they significantly affect the color formation of raw sugar. This study investigated the relationship between color formation and phenolic compounds during a Very High Polarization (VHP) sugar manufacturing process, in which the RB92579 genotype represents about 50% of the processed sugarcane. The products evaluated during the industrial processing of sugarcane were: raw juice, mixed juice, lime-treated juice, clarified juice, syrup, massecuite, and VHP sugar. The polyphenols catechin (CAT), chlorogenic acid (CGA), caffeic acid (CAF), vanillin (VAN), syringaldehyde (SYR), p-coumaric acid (p-COU), coumarin (CUM), and rutin (RUT) were quantified by high-performance liquid chromatography (HPLC). The highest concentrations of CGA and SYR were obtained from the sucrose crystallization product (massecuite), similarly to the parameters of color, total phenols and the total polyphenol content. CGA was the predominant polyphenol in the samples of clarified juice, syrup, massecuite and VHP sugar, with the latter presenting concentrations above 50%. The presence of phenolic compounds provided different indices of color during the production process. In this context, chlorogenic acid (CGA) was the compound that presented the most expressive results, contributing significantly to the formation of color in sugarcane processing products, which is a fact that has not yet been reported in the literature. The color of the VHP sugar crystals also had a positive relationship with the concentration of phenolics, with greater evidence for CGA.

## Introduction

Global sugar production approaches 200 million tons, with Brazil being the largest producer with approximately 20% of this total^1^. VHP (Very High Polarization) sugar has been used in several countries as a raw material for the production of refined sugar or in other industrialization processes^2^.

In recent decades there has been a significant increase in the supply of sugarcane genotypes with excellent agricultural and industrial productivity. However, some of these genotypes bring with them compounds that add color to the juice, negatively interfering with the factory's quality control. In Brazil, the genotype RB92579 marketed by RIDESA (REDE INTER UNIVERSITÁRIA PARA O DESENVOLVIMENTO DO SETOR SUCROENERGÉTICO) has already reached the third position of cultivation in Brazilian sugarcane fields in less than 20 years after the beginning of its commercial cultivation, with more than 487 thousand hectares planted^3^. However, this genotype offers an extremely dark juice with a high content of phenols, making it difficult to produce lower colored sugar^4^.

The sugar manufacturing process begins with the extraction of the juice, which is carried out through mills or diffusers. The extracted juice is treated at 98–105 °C by using chemicals (lime, sulfur dioxide, phosphoric acid, and flocculant) for the subsequent separation of impurities in the clarifier^5^. The next stage is evaporation, in which the clarified juice is concentrated from approximately 15 to 60 °Brix, thus obtaining the syrup, a high viscosity product that is concentrated in the cooking stage, promoting the formation of sucrose crystals. At the end of crystallization, the cooked dough is cooled and sent to the sugar centrifuges, in which the separation between sugar crystals and molasses occurs^6^.

Color is one of the main factors of sugar quality, being an important attribute used to determine the price in its commercialization^7,8^. Removing the color generated from the processing of raw sugar represents a high cost for the sugar refining industry, thus being one of the main bottlenecks of the industrial process^9^. The chemical components responsible for the darkening of sugar are found in the raw material itself (sugarcane) or originated during the production process. The amount of coloring matter represents about 17% of the organic non-sugars in the sugarcane juice^10^. The main colorants found in the sugarcane are: chlorophylls, carotenoids, xanthophylls, and flavonoids^11^. The first three compounds are insoluble in water and are easily separated during juice clarification. Phenolic acids, flavonoids and other phenolic compounds are low molecular weight plant pigments that are present in sugarcane and play an important role in the formation of sugar color^12^. Melanins, melanoidins, caramels, and alkaline degradation products of hexoses are also formed during sugar production^13^.

Among the phenolic compounds presented in the sugarcane juice are flavonoids (derivatives of apigenin, luteolin, and tricine) and phenolic acids, mainly caffeic, synaptic, and chlorogenic acids^14^. The main polyphenol found in sugarcane fiber is lignin, which consists of an aromatic system composed of phenylpropane units that will lead to the formation of phenols, acids, and aromatic aldehydes (vanillin and syringaldehyde)^15^.

Some studies show significant content of phenolic compounds in the products of sugarcane processing, but none of them show concrete relationships between these substances and the formation of color. Payet et al.^16^ found high concentrations of ferulic and p-coumaric acids in the clarified juice and syrup, representing 82 and 70% of the phenolic compounds determined in these products, respectively. Nguyen and Doherty^17^ quantified chlorogenic acid in large amounts in raw and burned sugarcane. The phenolic profile of the sugarcane varieties studied by Duarte-Almeida et al.^18^ was predominantly composed of cinnamic acids (caffeic, chlorogenic, p-coumaric and ferulic), with emphasis on chlorogenic acid, which was the substance found in the highest concentration. Ogando et al.^19^ used electrocoagulation in the sugarcane juice clarification process and found that the concentrations of benzoic acids (gallic, p-hydroxybenzoic, vanillic and syringic), hydroxycinnamic acids (caffeic, p-coumaric and ferulic) and flavones (naringenin and quercetin) were not affected by the treatment, and consequently, showed no correlation with the color of the juice.

The search for a more efficient and lower cost color removal process for VHP sugar represents a challenge for the sugar industry, requiring a better understanding of the behavior of compounds that add color throughout the production stages, such as polyphenols. In view of the above, this study investigated the influence of phenolic compounds on the color formation of sugarcane processing products for the production of VHP sugar, in a process with predominance of the genotype RB92579.

## Materials and methods

### Samples

**Figure 1:**
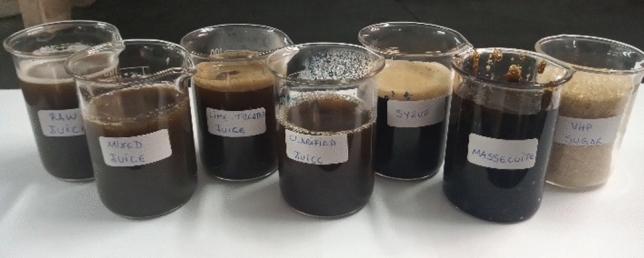
Major products obtained from the VHP sugar manufacturing process: raw juice, mixed juice, lime-treated juice, clarified juice, syrup, massecuite, and VHP sugar.

Samples from six stages of the VHP sugar manufacturing process were collected from an industrial unit located in the Zona da Mata region, state of Alagoas, Brazil. All collections took place with due authorization from the aforementioned industry. This industry uses approximately 30 varieties of sugarcane, in which RB92579 represents about 50% of this total. The collection period took place from October 2019 to March 2021, with a total of 48 samples acquired for analysis. All samples were named according to the product obtained from the different stages of the process: raw juice, mixed juice, lime-treated juice, clarified juice, syrup, and massecuite (Fig. 1). The raw juice, from the sampling probe, was obtained from freshly crushed cane^20^; while mixed juice, lime-treated juice, clarified juice, syrup, and massecuite were obtained at the end of the extraction, liming, clarification, evaporation, and crystallization processes, respectively. VHP sugar was collected in six different colors, under the same production process conditions. Samples of VHP sugar were obtained once every 30 days during the 2020/2021 harvest season as follows: 100 g of VHP sugar were collected every 2 h over a period of 6 h. At the end, the whole amount was mixed, obtaining a mix of 400 g.

### Reagents

The solvents used were: HPLC methyl alcohol (J. T. Baker), formic acid (Dynamics), and ultrapure water obtained from a Milli-Q system. Gallic acid, catechol, catechin, chlorogenic acid, caffeic acid, vanillin, syringaldehyde, p-coumaric acid, coumarin, rutin, myricetin, and quercetin were acquired by the Sigma-Aldrich Brasil LTDA. All other reagents were of analytical grade.

### ICUMSA color test

The International Commission for Uniform Methods of Sugar Analysis (ICUMSA) color intensity was obtained using an adaptation of the GS 2/3-9 and GS 1/3-7 methods^21,22^. For juice, syrup, and massecuite, the Brix degree of the samples was read for further dilution, promoting its correction to 5 °Brix. The diluted material was vacuum filtered using an AP25 pre-filter and a filter membrane (0.45 µm porosity), and the filtrate was pH-corrected to 7 ± 0.1 with HCl (0.05 M) and NaOH (0.05 M). Finally, a new Brix measurement was taken for subsequent reading on the spectrophotometer, configured at a wavelength of 420 nm and measured in a 1 cm cuvette at 0.0 absorbance and 100% transmittance with distilled water. The color is expressed in ICUMSA units (IU) and determined using Eq. (1).

1 $$\mathrm{Cor }\left(\mathrm{IU}\right)=\frac{\mathrm{ABS}}{\mathrm{b}.\mathrm{c}}\times 100.$$

In which: ABS: Absorbancy of the solution. b: the optical path length (cm). c: Filtrate concentration (g/mL) (using the Brix value).

For VHP sugar, 20 g of sample was diluted in a 100 mL flask with the addition of 10 mL of the MOPS buffer solution (4-morpholino propane sulfonic acid). Subsequently, the samples were filtrated and the Brix measure was taken for the spectrophotometric reading, following the same protocols as described for juices, syrup, and massecuite. Each assay was performed in duplicates with the results expressed in IU.

### Total phenols

The total phenols were determined using the Folin-Ciocalteu^23^ method, with some modifications. The calibration curve was prepared using galic acid solutions in concentrations between 2 and 10 mg/L. In the quantification of phenols, 2 g of sample were solubilized and transferred to a 50 mL volumetric flask, completing it with distilled water. A 2 mL aliquot of the diluted sample was mixed with 0.4 mL of the Folin-Ciocalteu reagent and 0.4 mL of the sodium hydroxide solution (7.0% v/v). After 60 min, the reading was performed with a spectrophotometer adjusted to 760 nm, using distilled water for the blank, which is prepared in the same way as the sample. Each test was performed in duplicate, with the results for juice, syrup and massecuite expressed in ppm/°Brix, while the results for sugar samples were expressed in mg/kg.

### Extraction

Samples of juice and syrup were previously centrifuged (2000 rpm) and filtered on filter paper (7 cm diameter). Then, the solubilization of 0.50 g of the samples (juice, syrup, and massecuites) was performed using 5.0 mL of cyclohexane in an ultrasound bath at 35 °C. The phenolic compounds were extracted using 15 mL aliquots of methanol: water (70: 30 v/v) divided into three sessions. The separation was carried out in a decantation flask, with the solvent being evaporated under vacuum in a rotary evaporator to an approximate volume of 2 mL. Finally, a new 0.45 μm polyethylene membrane filtration (Millipore) was carried out and the extract was placed in a 1.5 mL vial for injection.

### Determination of phenolic compounds

Concentrations of gallic acid (GAA), catechol (COL), catechin (CAT), chlorogenic acid (CGA), caffeic acid (CAF), vanillin (VAN), syringaldehyde (SYR), p-coumaric acid (p-COU), coumarin (CUM), rutin (RUT), myricetin (MIR), and quercetin (QUE) were determined by High Performance Liquid Chromatography (HPLC) in a Shimadzu chromatograph equipped with the following components: an automatic sampler (SIL-20A), pump (LC-20A), oven (CTO-20A), UV/VIS detector (SPD-20A), Shim-pack CLC-ODS (M)^®^ C18 column (4.6 × 250 mm) (particle size of 5 µm), and controlled by LC-Solution 1.0 software.

The experimental conditions were configured for a flow of 0.6 mL/min, analysis time of 80 min, injection volume of 20 µL and oven temperature of 33 °C, with the mobile phase consisting of solvents A (formic acid solution 0.1%) and B (methanol), where samples and standards were eluted according to the gradient of variation: from 0 to 15 min (7–25% B); 15 to 38 min (25–50% B); 38 to 58 min (50–85% B); 58 to 62 min (85–25% B); 62 to 80 min (25–7% B). The identity of the analytes was confirmed by the retention time of the sample peaks in comparison with the chromatogram of the analytical standards. Each test was performed in duplicate, with the results for juice, syrup and massecuite expressed in ppm/°Brix, while the results for sugar samples were expressed in mg/kg.

### Statistical analysis

The data were evaluated using the Duncan test for comparison purposes with a significance level set at 5%.

## Results and discussion

The influence of phenolic compounds on color formation was assessed based in two situations: during the VHP sugar manufacturing process as well as for different samples of VHP sugar.

### VHP sugar production

To transparently and objectively represent the behavior of color and phenolic compounds, six products from the VHP sugar manufacturing process were chosen for monitoring, namely: raw juice, mixed juice, lime-treated juice, clarified juice, syrup, massecuite, and VHP sugar. In all samples, Brix, ICUMSA color, total phenols (Folin-Ciocalteu), and polyphenols (HPLC) were obtained. Due to the significant variation in solids concentration (°Brix) of the products during the manufacturing stages, the quantification of total phenols and polyphenols were expressed in ppm/ºBrix.

### °Brix and ICUMSA color

**Figure 2:**
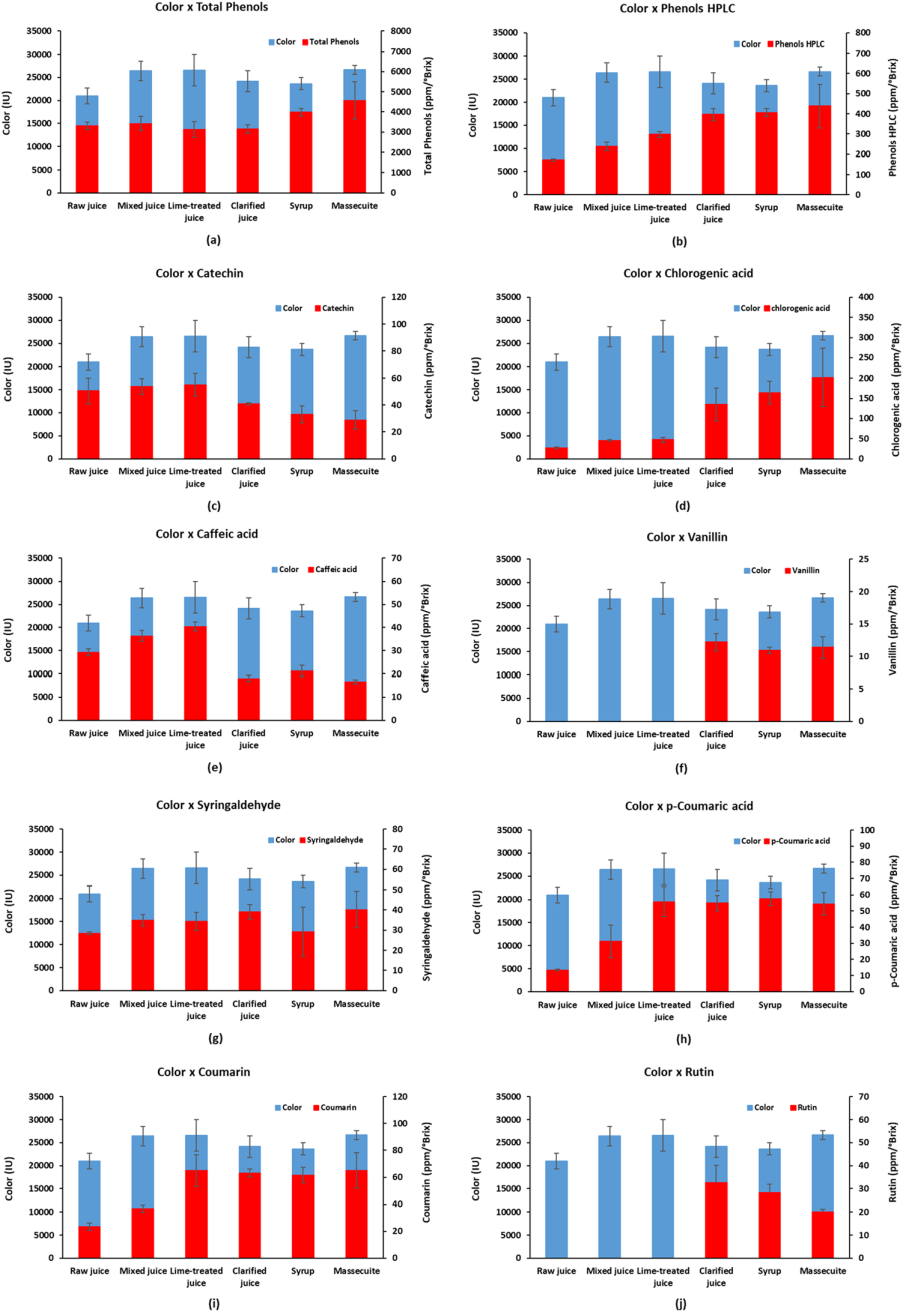
Relationship between color of sugarcane products and: (**a**) total phenols (**b**) total polyphenol content (**c**) catechin (**d**) Chlorogenic acid (**e**) caffeic acid (**f**) vanillin; (**g**) syringaldehyde; (**h**) p-coumaric acid; (**i**) coumarin (**j**) rutin.

The raw juice represents the raw material of the production of VHP sugar, which was obtained from individual samples of sugarcane collected before processing (sampling probe). Among the analyzed products, the raw juice presented the lowest color indexes (Fig. 2). This fact can be explained due to the individualization of the samples, which depend on factors related to the characteristics of sugarcane, such as: genotype, climatic conditions, maturation and type of harvest.

Figure 2 shows that the color of the mixed juice (juice obtained from the mills) was superior to that of the raw juice (p < 0.05), meaning that, after extraction, the juice darkened. However, there was a significant decrease (p < 0.05) in the soluble solids content (°Brix) (Table 1) caused by the imbibition process carried out in the mills.

**Table 1:** Results of °Brix for the VHP sugar manufacturing stages.

Stages	°Brix
Raw juice	20.7 ± 1.3^b^
Mixed juice	14.0 ± 0.4^c^
Lime-treated juice	13.7 ± 0.1^c^
Clarified juice	14.7 ± 0.3^c^
Syrup	61.4 ± 3.2^a^
Massecuite	93.5 ± 0.4^a^

^a,b,c,d,e^Values with different superscript letters within the same columns are significantly different (p < 0.05) pelo teste de Duncan.

The browning of the mixed juice occurs due to the enzymatic oxidation of phenolic compounds by the action of polyphenol oxidase enzymes (PPO), resulting in the formation of quinones and, after condensation, highly colored polymers^24^. This reaction occurs from the rupture of the sugarcane plant tissues in the mills and is catalyzed by the presence of metals such as iron.

Table 1 and Fig. 2 shows that the lime-treated juice (juice obtained after adding lime) had no significant difference (p > 0.05) in color, °Brix, and total phenols compared to the mixed juice. The main parameter to be controlled in this juice is the pH, whose value must not exceed 8.0 to avoid the formation of color by the Maillard reaction and not be lower than 6.0 to avoid sucrose inversion.

The clarified juice was obtained from a trayless SRI (Sugar Research Institute) clarifier, which works with a fully automated control system with short juice retention times (approximately 40 min). The color of the clarified juice (24,140 IU) is the result of the elimination of insoluble impurities, mainly high molecular weight dyes. The color intensity of the clarified juice can be influenced by the quality of the sugarcane and the clarification process itself. Stability in temperature and pH, shorter juice retention time in clarifiers and correct addition of chemicals are factors that favor no color formation during juice clarification^25^. The clarification step is crucial for the quality control of the VHP sugar that will be produced, because any impurity contained in the clarified juice will hardly be removed in the subsequent steps.

The syrup (final product of the evaporation process of the clarified juice) presented a high level of soluble solids (°Brix) due to the elimination of a large volume of water contained in the juice, but had no significant difference (p > 0.05) in color compared to the clarified juice (Fig. 2). According to Mersad et al.^13^, the caramelization (process of thermal degradation of sucrose) of the juice, still in the first effect, can increase the color of the syrup during the evaporation process due to high temperatures (above 180 °C). According to Shah^26^, some important factors to minimize color formation during the evaporation process are: temperature, juice circulation, retention time and concentration levels. Campiol et al.^27^ managed to reduce the color of the VHP sugar syrup by 82% using hydrogen peroxide (H_2_O_2_) solution (35%, v/v) at pH 7.5 and a temperature of 50 °C.

Crystallization is the process in which sucrose crystals form and grow. The product formed at this stage is called massecuite. The massecuite has a high concentration of solids (93.5°Brix) (Table 1) and high color intensity (26,660 IU) (Fig. 2). Booysen and Davis^28^ state that crystallization is the most critical process for color control in the sugarcane factory, because it will depend both on the quality of the syrup to be processed and on the control of operational parameters, such as: mass circulation, temperature and retention time.

### Total phenols and total polyphenol content

The total phenols content ranged from 3130 ppm/°Brix (limed-treated juice) to 4572 ppm/°Brix (massecuite), showing significant increase levels (p < 0.05) with products with higher sugar concentration (syrup and massecuite) (Fig. 2a). Not considering the relationship with the solids concentration (°Brix), the total phenols content of the raw juice and syrup would be 685 and 2446 mg/L, respectively, which are values close to the 728 mg/L and 1988 mg/L reported by Eggleston^29^.

The total content of polyphenols, which were determined by HPLC, increased throughout the production process, highlighting the increase of 33% from the lime-treated juice to the clarified juice (Fig. 2b).

In all stages analyzed, the amount of total phenols was greater than the total of polyphenols, ranging from 5.2 to 12.6%, which makes it evident that there is a significant amount of phenolic compounds in the analyzed products (Fig. 2a,b). Furthermore, the Folin-Ciocalteu method measures all compounds (phenolic or not) that oxidize under test conditions, overestimating the results^30^.

### Phenolic compounds

**Figure 3:**
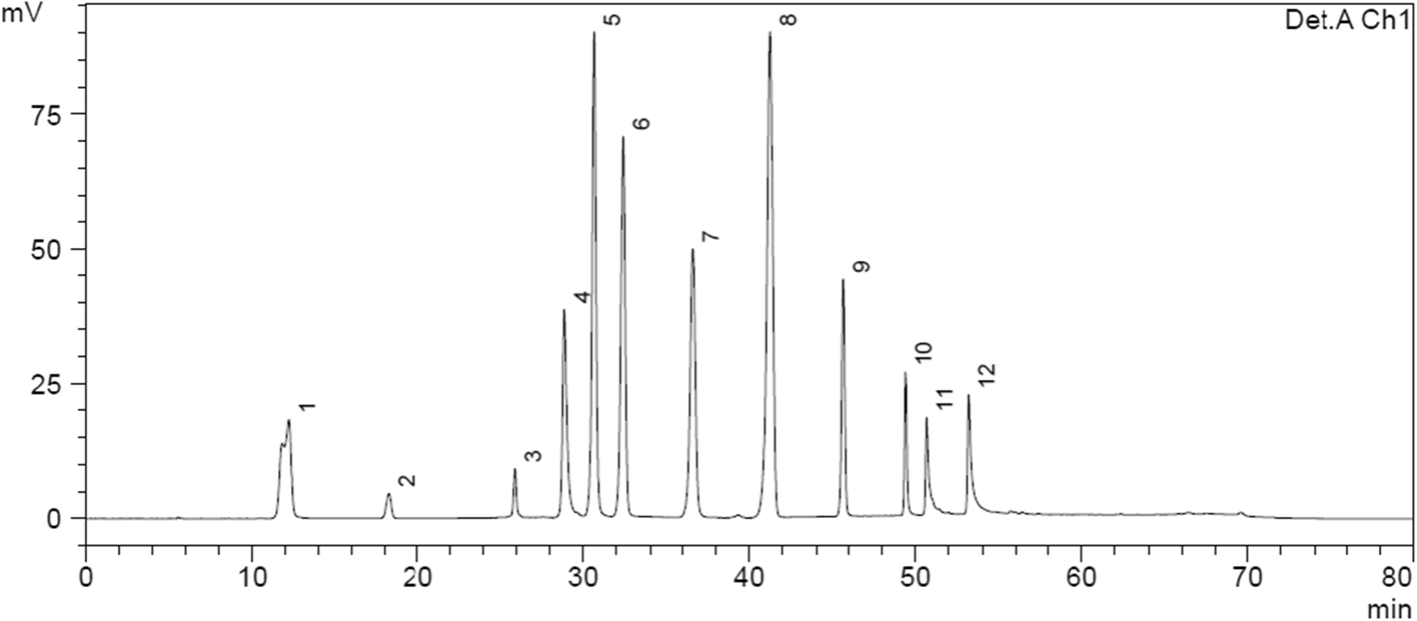
Chromatogram of analytical standards: (1) gallic acid; (2) catechol; (3) catechin; (4) chlorogenic acid; (5) caffeic acid; (6) vanillin; (7) syringaldehyde; (8) p-coumaric acid; (9) coumarin; (10) rutin; (11) myricetin and (12) quercetin.

Eight phenolic compounds were identified from the chromatogram of analytical standards (Fig. 3): catechin (CAT), chlorogenic acid (CGA), caffeic acid (CAF), vanillin (VAN), syringealdehyde (SYR), p-coumaric acid (p-COU), coumarin (CUM) and rutin (RUT).

VAN and RUT were not identified in the steps before juice clarification (raw, mixed, and lime-treated juices) (Fig. 2f,j). VAN was identified in the clarified juice, probably due to the decomposition of the lignin presented in the fiber particles contained in the lime-treated juice, under alkaline conditions and heating^31^. The decrease of the RUT content (p < 0.05) after obtaining the syrup may be attributed to hydrothermal decomposition (temperature above 120 °C) caused by the evaporation process^32^.

In all stages of VHP sugar processing, the CUM content was higher than p-COU (Fig. 2h,i). For these two compounds, there was a significant increase (p < 0.05) in the concentration of the raw juice until the lime-treated juice, with an increase of 175 and 315% for CUM and p-COU, respectively. After obtaining the clarified juice, the CUM content decreased in the syrup and, subsequently, increased in the massecuite, while the opposite occurred for the p-COU, both without significant difference (p > 0.05). The concentrations of CUM identified in the clarified juice, syrup and massecuite were expressive, with only CGA being lower. Payet et al.^16^ did not identify CUM in the sugar manufacturing stages; however, high concentrations of p-COU in the clarified juice and syrup were found, representing 54 and 39% of the phenolic compounds determined in these products, respectively.

The concentrations of CAT (p > 0.05) and CAF (p < 0.05) showed an increase during the first stages of the process (raw, mixed, and lime-treated juices) and a sharp drop in the following steps (clarified juice, syrup, and massecuite) (Fig. 2c,e). This behavior was more evident and significant with CAF in the clarification stage, where a reduction of more than 55% in its concentration was found compared to the previous stage.

**Figure 4:**
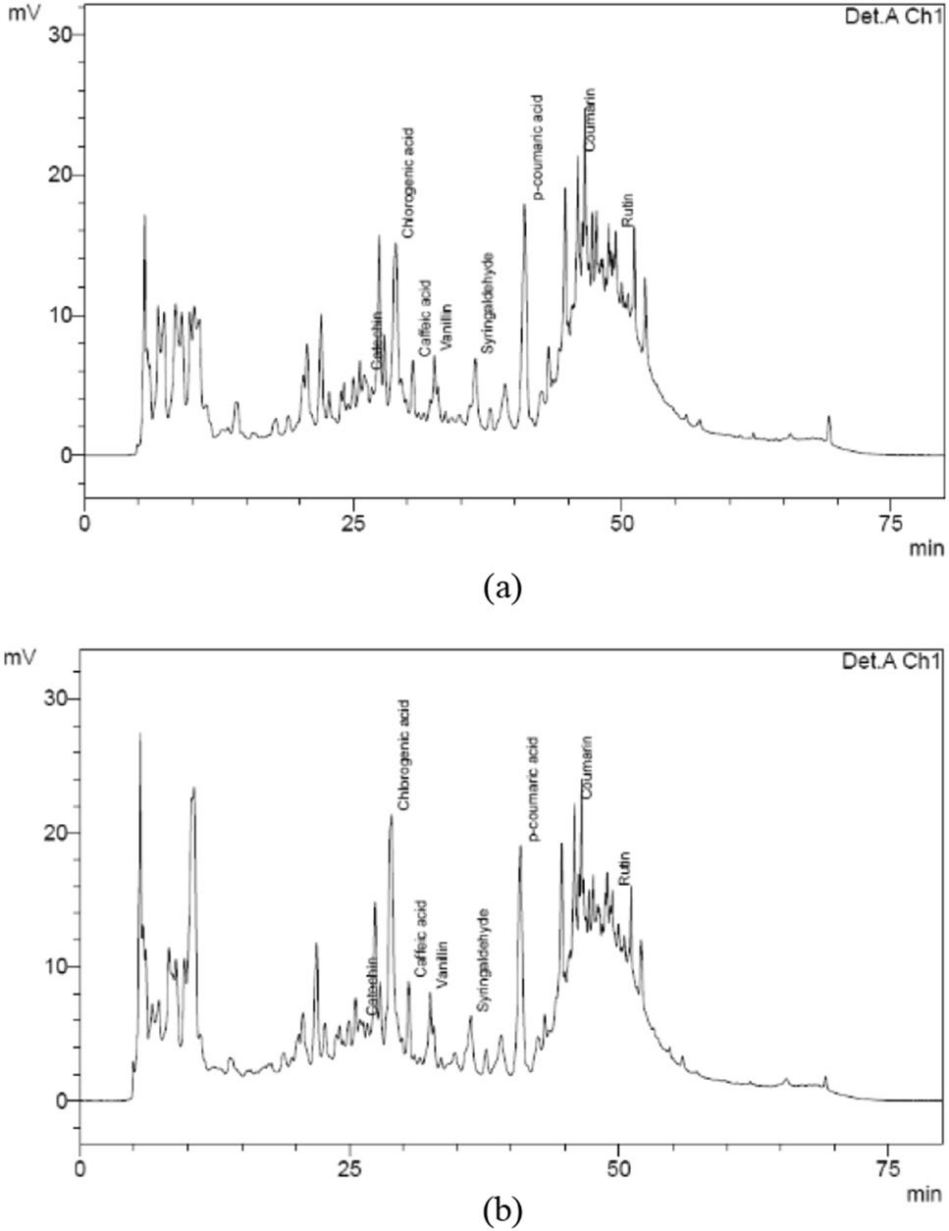
Chromatograms of the products obtained from the VHP sugar manufacturing stages: (**a**) syrup and (**b**) massecuite.

SYR and CGA were the only phenolic compounds that increased their concentrations after obtaining the clarified juice, mainly CGA with 187% increase (Fig. 2d,g). A similar increase (166%) was obtained by Paton^33^, who related the CGA concentration of the clarified and mixed juices. CGA was predominant in the last stages of production, representing 40% of the total phenolic compounds quantified in the syrup, which is higher than the 19% found by Duarte-Almeida et al.^18^, who also evaluated the VHP sugar manufacturing process, but did not determine the color. The highest indexes of CGA and SYR were obtained from the massecuite, in the same way as the color, total phenols, and total polyphenol content. Figure 4 shows the chromatograms obtained from syrup and massecuite, which showed the highest concentration of chlorogenic acid.

Regarding the clarified juice, the contrast between the significant increase in the CGA content and the abrupt reduction in the CAF content was evident (Fig. 2d,e), which may be explained by the formation of CGA. The most common and the only commercially available chlorogenic acid is 5-*O*-caffeoylquinic acid (5-CQA), which is formed by esterifying caffeic acid (3,4-hydroxycinnamic acid) with quinic acid (1 L^−1^ (OH), 3,4/5-tetrahydroxycyclohexane carboxylic acid)^34,35^. The hypothesis that this reaction occurred in the clarification stage was considered for some reasons: the sugarcane juice has quinic acid^36^; the pH close to neutrality and the high temperature (105 °C) favor the reaction^37^; and calcium added to the juice in the form of Ca(OH)_2_ in the liming step is proven to be a good catalyst^38,39^. Therefore, it may be inferred that the juice clarification stage promoted the formation of CGA by the degradation of CAF.

### The relationship between color and phenolic compounds

Figure 2 shows the relationship between color and phenolic compounds in the main products of the VHP sugar manufacturing stages. Three significant variations (p < 0.05) in color were observed throughout the industrial process: an increase in the mixed juice, a decrease in the clarified juice and another increase in the massecuite.

The increase in color of the mixed juice in relation to the raw juice was the highest among all the analyzed steps (26.0%) (Fig. 2) and also the only one that showed an increase in the concentration of all identified polyphenols, especially p-COU (Fig. 2h). In addition to the enzymatic browning mentioned previously, the juice extraction step promotes the simultaneous use of sugarcane in the most diverse varieties and characteristics, and this may reflect on these quality parameters.

The color reduction promoted by the clarified juice was 9.2% in relation to the lime-treated juice (Fig. 2). Booysen and Davis^28^ reported that this discoloration can reach 20–25% during the clarification process. In this step, there was a significant increase in the content of polyphenols, due to the growth of CGA (Fig. 2d), SYR (Fig. 2g) and also by the quantification of VAN and RUT, which had not occurred in the previous steps (Fig. 2f,j). Figure 2g shows that SYR had a profile similar to that of color formation, highlighting the increase in concentration in the mixed juice and massecuite.

The formation of color in the massecuite (12.8%) compared to the syrup (Fig. 2) followed the increase in total phenols (14.7%), total polyphenol content (7.8%), CGA (22.7%) and SYR (37.6%), as shown in Fig. 2a,b,d,g, respectively. VAN had an insignificant increase in the crystallization step. It can be observed that as the juice was concentrated (°Brix increase) during the VHP sugar processing, the CAT and CAF contents decreased significantly, with their lowest indices obtained in the massecuite, the opposite behavior to the color (Fig. 2c,e). The CUM and p-COU profiles (Fig. 2h,i) were similar, with expressive concentrations in the last stages, but without significant change.

### VHP sugar

**Figure 5:**
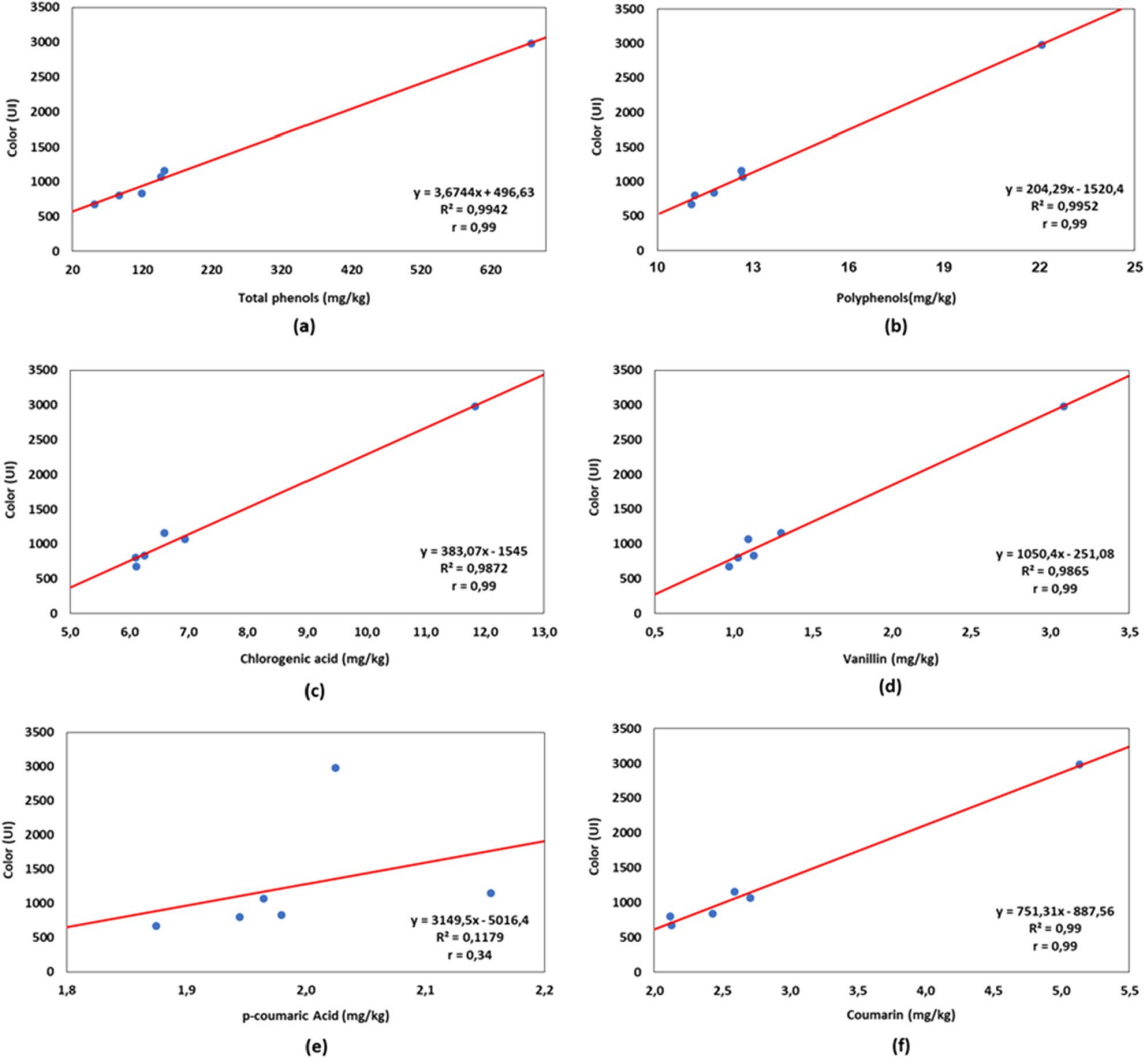
Correlation between VHP sugar color and: (**a**) total phenols; (**b**) total polyphenol content; (**c**) chlorogenic acid; (**d**) vanillin; (**e**) *p*-coumaric acid; (**f**) coumarin.

The analysis of VHP sugar were performed from six samples with different color intensities distributed in ascending order (Fig. 5). The high color range studied (675–2981 IU) (p < 0.05) was considered because, during storage, VHP sugar can undergo significant variations^40^. The color of the VHP sugar produced can vary based on three main factors: quality of raw material, type of juice treatment and specifications desired by buyers.

The concentration of phenolic compounds in VHP sugar (Fig. 5) was much lower compared to the production steps (Fig. 2), which may be explained by the fact that these substances are added into molasses, eliminated in the sugar centrifuges, leaving only a thin layer that surrounds the faces of the crystals. The concentration of total phenols ranged from 51 to 679 mg/kg, values similar to those found by Simioni et al.^41^, who evaluated 14 samples of VHP sugar with an ICUMSA color range between 270 and 1240 IU and total phenols between 78 and 260 mg/kg.

**Figure 6:**
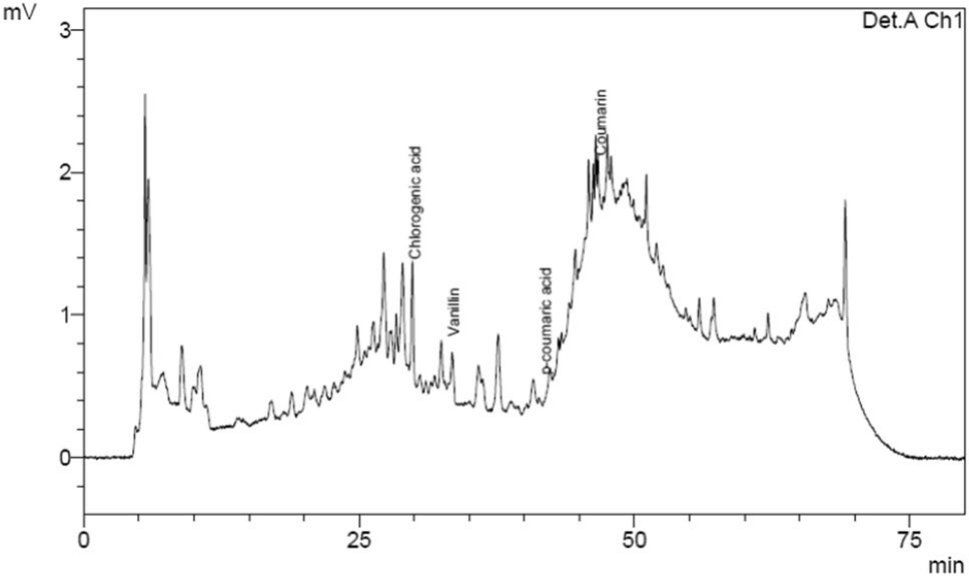
VHP sugar chromatogram.

The following phenolic compounds were found in the VHP sugar (Fig. 6): CGA, VAN, p-COU, and CUM. As reported by Bento^12^, phenolic acids (CGA and p-COU) are difficult to remove during extraction and refining, reaching the sugar crystals. CGA was the compound that presented the highest concentration in all samples, with values greater than 50% of the polyphenol composition. The concentrations of p-COU and CUM were similar in the first two samples, but from sugar crystals with a more accentuated color, there was a greater difference in the values due to the increase in the CUM content and no growth of p-COU, reaching the last sample with a difference of 153% between the two (p < 0.05). Although VAN was the phenol that showed the lowest concentrations in the first five samples, its growth was the most significant (137%) (p < 0.05) when the color of the sugar changed from 1156 to 2981 IU. Payet et. al. quantified the polyphenols present in brown sugar samples, not identifying any flavonoids and highlighting VAN and p-COU as the phenolic compounds contained in this type of sugar.

In Fig. 5, the correlation between color, total phenols, polyphenols, and CUM, VAN, and CGA phenols showed a good fit to the linear equation, with coefficients of determination (R^2^) greater than 0.98. Figure 5b–d,f also shows a strong positive correlation of color with total polyphenols by HPLC analysis, CGA, VAN, and CUM, presenting r = 0.99, whereas p-COU did not show significant correlation (r = 0.34) (p > 0.05) (Fig. 5e).

## Conclusions

The results of this study present an important and innovative analysis regarding the evaluation of the concentration of phenolic compounds in the color formation of the products formed in the stages of VHP sugar production, in a process with predominance of the genotype RB92579. At all stages of the process, there was variation in the composition of polyphenols, with emphasis on clarification of the juice. SYR showed a positive relationship with color in the manufacturing stages, despite not being identified in VHP sugar. CGA, CUM and VAN showed significant interference in the color increase of the darker VHP sugar crystals. Chlorogenic acid (CGA) proved to be the phenolic compound with the greatest influence on color formation during the processing of sugarcane for the manufacture of VHP sugar, due to the strong relationship with color and high concentrations in the final product and in the production stages. The results of this study suggest that the phenolic profiles obtained may represent an interesting tool in the development of new technologies associated with color removal in the sugar industry.

## Data Availability

The datasets generated during and/or analyzed during the current study are available from the corresponding author on reasonable request.

## References

[1] USDA. United States Department of Agriculture. Foreign Agricultural Service. https://apps.fas.usda.gov/newgainapi/api/Report/DownloadReportByFileName?fileName=Sugar%20Annual_Sao%20Paulo%20ATO_Brazil_04-15-2021.pdf (2021).

[2] Morilla CHG, Alves LRA, Aguiar CL (2015). Sulfitation sugarcane juice clarification process: Trade barriers and economic impacts. A Economia em Revista..

[3] Oliveira, R. A., Barbosa, V. G. S. & Daros, E. 50 years of RB sugarcane varieties: 30 years of RIDESA. Curitiba: UFPR. *RIDESA*. (2021).

[4] Azevedo ACB (2019). Enzymatic polyphenoloxidase inactivation with temperature and ozone in sugarcane variety RB 92579 to produce lower color sugar. Braz. J. Food Technol..

[5] Li W (2017). Pilot demonstration of ceramic membrane ultrafiltration of sugarcane juice for raw sugar production. Sugar Tech..

[6] Lopes, C. H. *Sugar of Cane Production Technology* 183. (EdUFSCAR, 2011).

[7] Oliveira TO, Esquiaveto MMM, Júnior JFS (2007). Sugar specification parameters and their impact on the food industry. Food Sci. Technol..

[8] Rein, P. *Cane Sugar Engineering* 943. (Bartens, 2012).

[9] Fang Y, Ellisa A, Uchimiyab M, Strathmanna TJ (2019). Selective oxidation of colour-inducing constituents in raw sugar cane juice with potassium permanganate. Food Chem..

[10] Rupa TR, Asokan S (2008). Effect of rind pigments and juice colorants on juice claribility, settling time and mud volume of sugarcane. Sugar Tech..

[11] Larrahondo, J. E. & Ordoñez, S. P. *Sugarcane Flavonoids* 40*.* (Valle, 2014).

[12] Bento LSM (2009). Colourants through sugar production and refining (Part 1). Sugar Ind..

[13] Mersad A, Lewandowski R, Heyd B, Decloux M (2003). Colorants in the sugar industry: Laboratory preparation and spectrometric analysis. Int. Sugar J..

[14] Duarte-Almeida JM, Novoa AV, Linares AF, Lajolo FM, Genovese MI (2006). Antioxidant activity of phenolics compounds from sugar cane (*Saccharum officinarum* L.) juice. Plant Foods Hum. Nutr..

[15] Miranda MB, Horii J, Alcarde AR (2006). Study of the effect of gamma irradiation (60co) on the quality of sugar cane spirit and on the cask of maturation. Food Sci. Technol..

[16] Payet B, Sing ASC, Smadja J (2006). Comparison of the concentrations of phenolic constituents in cane sugar manufacturing products with their antioxidant activities. J. Agric. Food Chem..

[17] Nguyen DMT, Doherty WOS (2011). Phenolics in sugar cane juice: Potential degradation by hydrogen peroxide and Fenton’s reagente. Proc. Aust. Soc. Sugar Cane Technol..

[18] Duarte-Almeida JM, Salatino A, Gevonese MI, Lajolo FM (2011). Phenolic composition and antioxidant activity of culms and sugarcane (*Saccharum officinarum* L.) products. Food Chem..

[19] Ogando FIB, Aguiar CL, Viotto JVN, Heredia FJ, Hernanz D (2019). Removal of phenolic, turbidity and color in sugarcane juice by electrocoagulation as a sulfur-free process. Food Res. Int..

[20] ABNT. Brazilian Association Of Technical Standards. Sugar cane—Extraction of juice by the automatic hydraulic press method and determination of weight of the moist cake. NBR16221. https://infostore.saiglobal.com/en-au/standards/abnt-nbr-16221-2019-776419_saig_nbr_nbr_2764466/ (2019).

[21] ICUMSA. International Comission For Uniform Methods of Sugar Analysis. Method GS2/3-9: The determination of sugar solution colour at pH 7.0. (Bartens, 2005).

[22] ICUMSA. International Comission For Uniform Methods of Sugar Analysis. Method GS1/3-7 Determination of the Solution Colour of Raw Sugars, Brown Sugars and Coloured Syrups at pH 7.0. (Bartens, 2011).

[23] Fonseca CR, Paiva JL, Rodriguez EM, Fernando JB, Teixeira ACSC (2017). Degradation of phenolic compounds in aqueous sucrose solutions by ozonation. Ozone Sci. Eng..

[24] Li H, Guo A, Wang H (2008). Mechanisms of oxidative browning of wine. Food Chem..

[25] Bourzutschky HCC (2005). Color formation and removal—Options for the sugar and sugar refining industries: A review (part 1). Sugar Ind..

[26] Shah S (2014). Some key principles for the design of Robert evaporators. S. Afr. Sugar Technol. Assoc..

[27] Campiol JLM, Magri NTC, Sartori JAS, Ogando FIB, Aguiar CL (2019). Color reduction of raw sugar syrup using hydrogen peroxide. Braz. J. Food Technol..

[28] Booysen KC, Davis SB (2019). Colourant behaviour during sugarcane processing. S. Afr. Sugar Technol. Assoc..

[29] Eggleston G (2018). Positive aspects of cane sugar and sugar cane derived products in food and nutrition. J. Agric. Food Chem..

[30] Payet B, Sing ASC, Smadja J (2005). Assessment of antioxidant activity of cane brown sugars by ABTS and DPPH radical scavenging assays: Determination of their polyphenolic and volatile constituints. J. Agric. Food Chem..

[31] Mota MIF, Pinto PCR, Loureiro JM, Rodrigues AE (2016). Recovery of vanillin and syringaldehyde from lignin oxidation: A review of separation and purification processes. Sep. Purif. Rev..

[32] Ravber M (2016). Hydrothermal degradation of rutin: Identification of degradation products and kinetics study. J. Agric. Food Chem..

[33] Paton NH (1992). The origin of colour in raw sugar. Proc. Aust. Soc. Sugar Cane Technol..

[34] Clifforf MN (1999). Chlorogenic acids and other cinnamates—Nature, occurence and dietary burden. J. Sci. Food Agric..

[35] Clifford MN (2000). Chlorogenic acids and other cinnamates—Nature, occurrence, dietary burden, absorption and metabolism. J. Sci. Food Agric..

[36] Glassop D, Roessner U, Bacic A, Bonnett GD (2007). Changes in the sugarcane metabolome with stem development. Are they related to Sucrose Accumulation?. Plant Cell Physiol..

[37] Alsalme A (2008). Probing the catalytic efficiency of supported heteropoly acids for esterification: Effect of weak catalyst. J. Chem..

[38] Demirbas A (2007). Biodiesel from sunflower oil in supercritical methanol with calcium oxide. Energy Convers. Manag..

[39] Gupta J, Agarwal M, Dalai AK (2017). Experimental evaluation of the catalytic efficiency of calcium based natural and modified catalyst for biodiesel synthesis. Int. J. Green Energy.

[40] Galati VC (2016). Quality of sugar stored in bulk in masonry silo. J. STAB..

[41] Simioni KR (2006). Effect of variety and harvest time on total phenol content in sugarcane. J. STAB..

